# Identification of endogenous normalizing genes for expression studies in inguinal ring tissue for scrotal hernias in pigs

**DOI:** 10.1371/journal.pone.0204348

**Published:** 2018-09-20

**Authors:** William Raphael Lorenzetti, Adriana Mercia Guaratini Ibelli, Jane de Oliveira Peixoto, Marcos Antonio Zanella Mores, Igor Ricardo Savoldi, Kamilla Bleil do Carmo, Haniel Cedraz de Oliveira, Mônica Corrêa Ledur

**Affiliations:** 1 Programa de Pós-graduação em Zootecnia, Centro de Educação Superior do Oeste (CEO), Universidade do Estado de Santa Catarina, UDESC, Chapecó, Santa Catarina, Brazil; 2 Embrapa Suínos e Aves, Concórdia, Santa Catarina, Brazil; 3 Universidade do Contestado, Concórdia, Santa Catarina, Brazil; 4 Universidade Federal de Viçosa, Viçosa, Minas Gerais, Brazil; Universitat de Lleida, SPAIN

## Abstract

The use of reference genes is required for relative quantification in gene expression analysis and since the stability of these genes could be variable depending on the experimental design, it has become indispensable to test the reliability of endogenous genes. Therefore, this study evaluated 10 reference candidate genes in two different experimental conditions in order to obtain stable genes to be used as reference in expression studies related to scrotal hernias in pigs. Two independent experiments were performed: one with 30 days-old MS115 pigs and the other with 60 days-old Landrace pigs. The inguinal ring/canal was collected, frozen and further submitted to real-time PCR analysis (qPCR). For the reference genes stability evaluation, four tools were used: GeNorm in the SLqPCR, BestKeeper, NormFinder and Comparative CT. A general ranking was generated using the BruteAggreg function of R environment. In this study, the *RPL19* was one of the most reliable endogenous genes for both experiments. The breed/age effects influenced the expression stability of candidate reference genes evaluated in the inguinal ring of pigs. Therefore, this study reinforces the importance of evaluating the stability of several endogenous genes previous their use, since a consensual set of reference genes is not easily obtained. Here, two sets of genes are recommended: *RPL19*, *RPL32* and *H3F3A* for 30-days MS115 and *PPIA* and *RPL19* for the 60 days-old Landrace pigs. This is the first study using the inguinal ring tissue and the results can be useful as an indicative for other studies working with gene expression in this tissue.

## Introduction

The real time PCR (qPCR) is one of the main approaches used for gene expression studies, being highly sensitive [1]. However, many factors related to this technique, since the quality of biological material up to the laboratorial procedures, might compromise the reliability of the qPCR results [2]. Furthermore, qPCR is a powerful technique to validate differentially expressed genes from global expression approaches, such as microarrays and, more recently, RNA-Seq [3]. Therefore, it is essential to standardize the methodologies to be used and, specifically considering gene expression studies using qPCR, the correct choice and use of reference genes, also known as endogenous genes, avoid mistaken results. The use of stable endogenous reference genes ensures that any variation in input RNA levels between samples is normalized, avoiding errors in the quantification [2]. Thus, knowing the behavior of these genes in each experimental design is crucial to obtain reliable results [1,4].

To be considered a valid reference gene its expression must not be variable between different experimental conditions, tissues or physiological state of the tissue or organism [2]. In relative gene expression analyses, the use of reference genes is required to normalize and obtain the fold-change, through mathematical algorithms, such as those described previously by Pfaffl (2001), Livak & Schmittgen (2001) and Schmittgen & Livak (2008) [5–7]. Some of the most well-known reference genes are *GAPDH* (glyceraldehyde 3-phosphate dehydrogenase), *PGK* (phosphoglycerate kinase), *UBQ (*ubiquitin), *RPL19* (ribosomal protein L19), *18S rRNA* (ribosomal RNA 18S), *β-actin* and *β-tubulin* [1] and they have been used in several studies, in many species, including pigs. However, the stability of the reference genes can be altered depending on the tissue, age, treatment and other conditions, which makes indispensable to test the stability of several genes before using those as reference [8–10]. Several studies searching for reliable endogenous genes in pigs have been reported [11–14] with different breeds, tissues and conditions. However, studies aiming to verify stable reference genes in the inguinal ring for scrotal hernia studies have not been reported to date.

The scrotal hernia is a malformation whereby intestinal loops traverse the abnormally open inguinal ring [15]. Although there are indications about the involvement of genetic components in the occurrence of this anomaly in humans [16,17] and other species [16], including the pig [18,19], the genes affecting this condition remain unknown. Therefore, expression studies are required to clarify the genetic mechanism involved in this malformation. Most of the expression studies searching for reference genes are based on muscular tissues with better characterized anatomy, such as *longissimus dorsi* [10,20–23] or even with a broader set of tissues [9,12,24]. No gene expression study is available in livestock using inguinal ring tissue, which is composed by connective and muscular tissues, and it is the site of occurrence of the scrotal hernia. Thus, knowing reliable reference genes for the inguinal canal is essential to obtain accurate gene expression assays with this tissue. Therefore, to obtain stable genes to be used as reference in expression studies related to scrotal hernias in pigs, 10 endogenous candidate genes were evaluated in the present study in two different experimental conditions.

## Materials and methods

### Animals and sample collection

This study was performed with the approval of the Embrapa Swine and Poultry Ethical Committee of Animal Use (CEUA) under the protocol number 011/2014. Two experiments were carried out to detect the best reference genes in two different ages: at 30 and 60 days of age. The details of each experiment are presented below:

#### Experiment 1 (E1)

Animals were raised at the Embrapa Swine and Poultry National Research Center farm until 30 days of age. A total of 18 entire male pigs of the MS115 synthetic line were used. The animals were grouped in normal (n = 9, absent from malformations and coming from litters with no history of hernias) and affected (n = 9, from litters with the presence of more than one animal with scrotal hernia).

#### Experiment 2 (E2)

Eight Landrace pigs with 60 days of age from the same nucleus farm, located in Santa Catarina State, Brazil, were used in this study. These animals were transported from the farm to the necropsy room at the Embrapa Swine and Poultry. As in the experiment 1, the animals were grouped in normal (n = 4) and affected (n = 4) with scrotal hernia.

For both experiments, animals were not related, i.e., they were chosen from different families in a case and control design, with cases and controls being from the same contemporary group. The euthanasia was performed by electrocution for 10 seconds, followed by immediate exsanguination, according to the practices recommended by the Ethics Committee. The necropsy was performed for the evaluation of possible problems and additional characteristics that could interfere in the accuracy of the data, as well as for the correct characterization of the hernia phenotype. Tissue samples from the inguinal ring/canal of normal and scrotal hernia-affected groups were collected and immediately frozen in liquid nitrogen and then stored at -80°C for subsequent RNA extraction. After necropsy and tissue collection, the piglets’ carcasses were destined for composting.

### RNA extraction

Tissue RNA extraction was performed according to the Trizol Reagent (Invitrogen, Carlsbad, CA) protocol. Samples containing about 100 mg of tissue were initially macerated in liquid nitrogen with mortar and pistil, properly treated for this procedure. After maceration, the generated contents were placed into 1.5 mL microtube containing 1 mL of the Trizol reagent, vortexed and then incubated for 5 minutes at room temperature (25 °C). Next, 200 μl of chloroform was added to the tube, shaken vigorously with the hands for 15 seconds, and finally incubated at room temperature for 5 minutes. After incubation, centrifugation was performed at 11,000 rpm (rotations per min) at 4 °C for 15 minutes. Thereafter, the aqueous phase was removed into a clean polypropylene tube and 500 μl of isopropanol was added. The tube was stirred and subsequently incubated for 10 minutes at room temperature. After 10 minutes, the tubes containing the sample were centrifuged for 10 minutes at 10,000 rpm at 4 °C. The supernatant was discarded and the pellet washed with 1 mL of 75% ethanol and homogenized in vortex. This was centrifuged at 9,000 rpm for 5 minutes at 4 °C. The supernatant was discarded and the pellet dried for 15 minutes at room temperature, resuspended in DEPC water and heated at 55 °C for 10 minutes. The quality and quantity of the total RNA were evaluated by spectrophotometer (Biodrop, UK) and also in 1% agarose gel. Finally, the total RNA extracted was conserved in ultrafreezer—80 °C.

### Complementary DNA (cDNA) synthesis

For the synthesis of complementary DNA (cDNA), the SuperScript III First-Strand Synthesis Supermix Kit (Invitrogen, USA) was used. For each 3μg of total RNA, 1μL of Annealing buffer, 1μL of oligo dT 0.5μg / μL and water until the volume was completed in 10μL were added, incubated at 65°C for 5 minutes and then cooled in ice for 1 minute. Then, 10 μL of 2X First-Strand reaction mix and 2 μL of SuperscriptIII/RNAseOUT enzyme mix (Invitrogen, USA) were added to the mixture, being incubated for 50 minutes at 50 °C and subsequently inactivated for 5 minutes at 85 °C, and then stored at -20 °C.

### Relative quantification using qPCR

The relative quantification of each putative reference gene was performed by qPCR. The expression pattern of the following genes was evaluated: hydroxymethylbilane synthase (*HMBS*), tyrosine 3-monooxygenase/tryptophan 5-monooxygenase activation protein zeta (*YWHAZ*), succinate dehydrogenase complex flavoprotein subunit A (*SDHA*), topoisomerase (*DNA*) II beta (*TOP2B*), ribosomal protein L13A (*RPL13A*), H3 histone, family 3A (*H3F3A*), eukaryotic translation elongation factor 1 alpha 1 (*EEF1A1*), ribosomal protein L32 (*RPL32*), ribosomal protein L19 (*RPL19*) and peptidyl prolyl cis-trans isomerase A (*PPIA*). The sequences and annotations for these 10 genes were obtained in the swine genome (*Sus scrofa*, v. 10.2) available in GeneBank (https://www.ncbi.nlm.nih.gov/genbank/) and Ensembl 86 (http://www.ensembl.org/index.html). Primers were designed in exon-exon junction regions, in order to avoid the genomic DNA amplification, using the Primer-Blast program [25] and are shown in Table 1. The qPCR reactions were carried out in duplicate in 15 μL final volume containing 1X of Maxima SYBR Green/ROX qPCR Master Mix (2X) (Thermo Fisher Scientific, USA), 0.05 to 0.13 μM of each primer and ~20 ng of cDNA. Reactions were performed in the Quantstudio 6 equipment (Thermo Fisher Scientific, USA) using SYBR Green as fluorescence dye with the following cycling condition: 95° for 10 min, 40 cycles of 15 seconds at 95°C and 30 seconds 60°C. In addition, the melting curve stage of 70°C to 95°C at 0.1°C/s for all genes studied were included to verify the primers specificity. The maximum allowed difference in Ct values between technical replicates was 0.3 Ct.

**Table 1:** Primers for the 10 reference candidate genes for the qPCR analysis in the inguinal ring of pigs.

Gene	Function	Primer Sequences (5’– 3’)	Ensembl ID
***HMBS*** hydroxymethylbilane synthase	Third enzyme of the biosynthetic pathway of the Heme group	F: AGGATGGGCAACTCTACCTGA R: ATGGATGGTGGCCTGCATAG	ENSSSCG00000015108
***RPL19*** ribosomal protein L19	Ribosomal protein 60S subunit component, L19E family	F: ACCGCCACATGTATCACAGTC R: TGTGCTCCATGAGAATCCGC	ENSSSCG00000017509
***RPL32*** ribosomal protein 32	Ribosomal protein 60S subunit component, L32E family	F: CAAAATTAAGCGGAACTGGCGG R: GCACATTAGCAGCACTTCAAGC	ENSSSCG00000027637
***EEF1A1*** eukaryotic translation elongation factor 1 alpha 1	Enzymatic delivery of aminoacyl tRNAs to the ribosome.	F: CCGCCAGGACACAGGT R: TTCCCATCTCCGCAGCCT	ENSSSCG00000004489
***H3F3A*** H3 histone, family 3A	3rd component of nuclear histones	F: CTTTGCAGGAGGCAAGTGAG R: TGGCATGGATAGCACACAGG	ENSSSCG00000023971
***RPL13A*** ribosomal protein 13A	Ribosomal protein 60S subunit component, L13A family	F: CCAAGCAGGTACTTCTGGGC R: GGCAGCATGCCTCGCA	ENSSSCG00000003166 ENSSSCG00000003167
***TOP2B*** topoisomerase (DNA) II beta	DNA transcription and replication	F: AGAAGAGCTGCTGCTGAAAGG R: TCCCCGTCATTTGTCACAGG	ENSSSCG00000011213
***SDHA*** succinate dehydrogenase complex flavoprotein subunit A	Encodes a major catalytic subunit of succinate-ubiquinone oxidoreductase, in the mitochondrial respiratory chain	F: TTGTACGGAAGGTCTCTGCG R: GATGACTCCACGACACTCCC	ENSSSCG00000020686
***YWHAZ*** tyrosine 3-monooxygenase/tryptophan 5-monooxygenase activation protein zeta	Regulation of signal transduction pathways through binding phosphoserine proteins	F: ATCAGATTGGGTCTGGCCCT R: GGTATCCGATGTCCACAATGTC	ENSSSCG00000006062
***PPIA*** peptidyl-prolyl cis-trans isomerase A	Accelerate the folding of proteins	F: GCGTCTCCTTCGAGCTGTTT R: ACTTGCCACCAGTGCCATTA	ENSSSCG00000016737

F: forward; R: reverse.

### Reference gene stability evaluation

A total of four algorithms widely used to identify the most stable expressed genes: the geNorm [26], NormFinder [27], BestKeeper [28] and Comparative ΔCt [29] were used to evaluate the reference candidate genes in the present study. The geNorm is a robust software that calculates an internal control gene-stability measurement (M) for each combination of two control genes tested, obtaining a transformed expression ratio and then, calculates a standard deviation of these pairwise gene combinations. The two most stable genes are determined based on the lowest M value, and values lower than 1.5 indicate stable genes [26]. The M values from geNorm were obtained using the SLqPCR package on R (http://bioconductor.org/packages/release/bioc/html/SLqPCR.html).

The NormFinder is a visual basic application for Microsoft Excel that calculates a stability value (S) based on intra and intergroup variation of genes tested, taking into account their co-regulation, ranking the genes according to their expression stability and similarity. The smallest S values indicate the best or the most stable genes to be used as normalizers [27]. In the NormFinder, the data used was transformed in log2, as suggested by the developer [27].

The BestKeeper is also an Excel-based tool for scoring the genes using an index (power of the gene) composed by the values of Ct, fold-change, standard deviation (SD) and coefficient of variation (CV) [28]. According to Pfaffl et al. (2004) [28], the most consistent genes will present values of SD of Cts lower than 1 and SD of X-Fold lower than 2. The authors also suggest not using genes with SD of Cts above 1.5 [28].

The Comparative ΔCt [29] uses a basic ΔCt approach to compare the relative expression of pairs of genes, creating a stability rank based on the ΔCt and average standard deviations. The genes with the lowest average SD and with constant ΔCt values are considered to be the most stable [29].

In addition, once all of the stability values for all tools were obtained, the BruteAggreg function, a weighted rank aggregation tool from the RankAggreg package [30] of R environment [31], that calculates a Spearman distance based on Monte Carlo algorithm, was used to determine a general ranking of the most stable genes for each experiment and analyzed tools (SLqPCR, NormFinder, BestKeeper and Comparative Ct). The BruteAggreg function was used twice for each experiment. This had to be done because the geNorm software ranks the two best genes at the same time. Then, these genes were both put in the 1^st^ and 2^nd^ positions for each experiment in BruteAggreg to improve the prediction of the best endogenous control gene.

## Results

The total RNA average concentration was 1,033.19 ng/μL for the normal and 1,052.66 ng/μL for the affected group in the Experiment 1, and 918.55 ng/μL for the normal and 995.03 ng/μL for the affected group, in the Experiment 2. Regarding the RNA quality, the average A260/280 ratio was 1.90 ± 0.04 and 2.06 ± 0.01 for the unaffected pig samples and 1.92 ± 0.05 and 2.07 ± 0.02 for herniated pig samples in the E1 and E2, respectively, evidencing a good quality of the RNA samples to be used in the further analysis.

**Fig 1:**
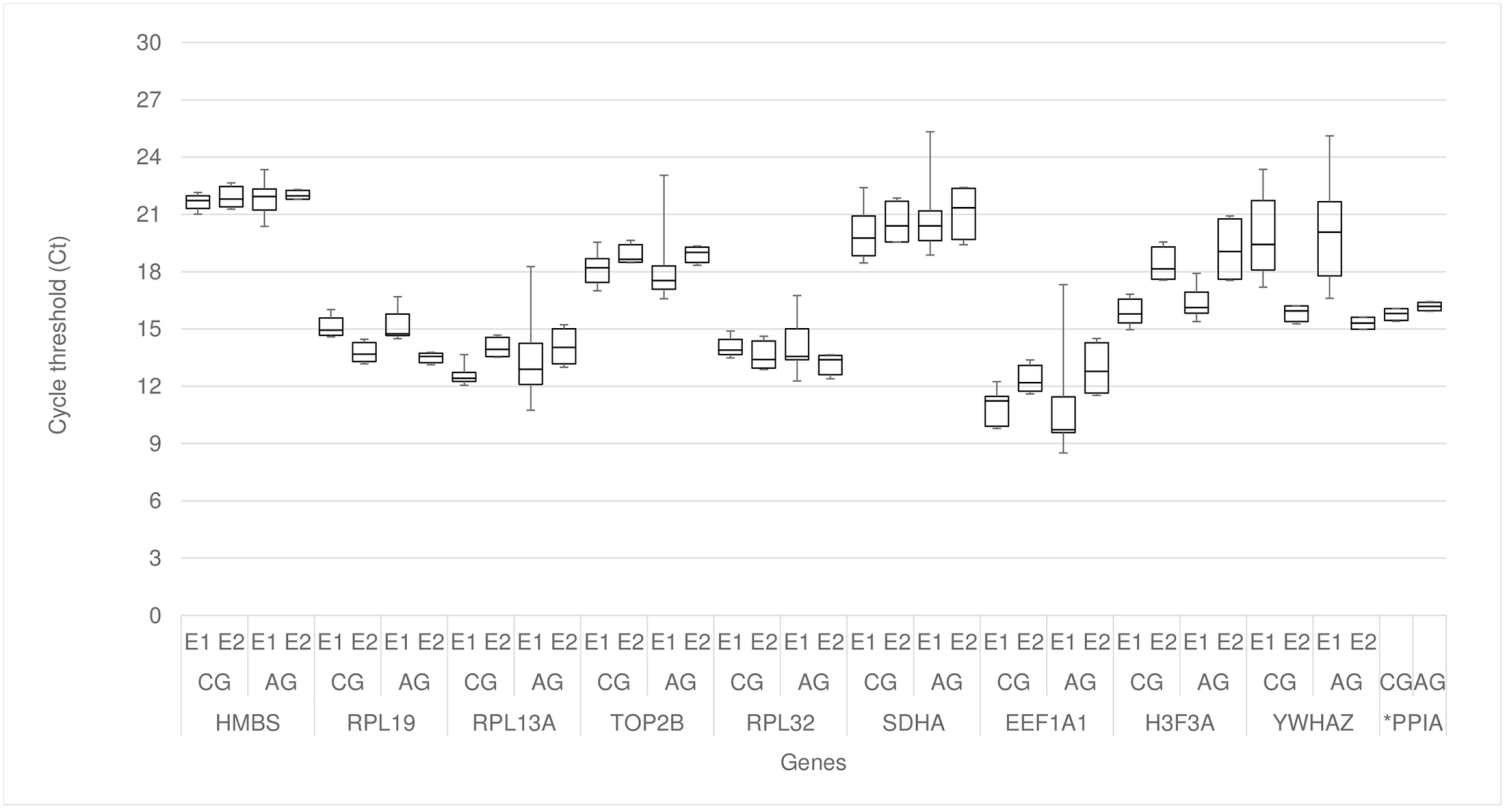
Cycle threshold (Ct) variation in normal and hernia-affected pigs in the two experiments. CG: control group; AG: affected group; 1 –experiment 1 and 2 –experiment 2. *PPIA: just the information about the experiment 2 was plotted, since there was no amplification for some samples in the experiment 1.

The mean Ct values (± SD) of the reference candidate genes ranged from approximately 10.5 to 22 (Fig 1, Table 2) according to each experiment. The *PPIA* gene was removed from the Experiment 1 analysis since there was no amplification for some of the samples, differing from the Experiment 2, where all samples amplified for this gene, with average Ct mean of 15.98 ± 0.34 and the smallest standard deviation.

**Table 2:** Average Ct means for the 10 reference candidate genes by group in each experiment.

Ct Mean ± SD						
	Experiment 1			Experiment 2		
Gene	Normal	Affected	Average	Normal	Affected	Average
**HMBS**	21.64 ± 0.39	21.82 ± 0.86	21.73 ± 0.66	21.89 ± 0.57	22.01 ± 0.25	21.95 ± 0.41
**RPL19**	15.12 ± 0.50	15.15 ± 0.80	15.14 ± 0.65	13.75 ± 0.53	13.51 ± 0.27	13.63 ± 0.41
**RPL13A**	12.56 ± 0.47	13.45 ± 2.14	13.00 ± 1.57	14.02 ± 0.53	14.07 ± 0.95	14.05 ± 0.71
**TOP2B**	18.15 ± 0.81	18.11 ± 1.95	18.13 ±1.45	18.85 ± 0.54	18.93 ± 0.43	18.89 ± 0.45
**RPL32**	14.05 ± 0.47	14.12 ± 1.31	14.09 ± 0.96	13.57 ± 0.76	13.20 ± 0.57	13.39 ± 0.65
**SDHA**	19.93 ± 1.32	20.80 ± 1.90	20.37 ± 1.45	20.55 ± 1.16	21.13 ± 1.43	20.84 ± 1.24
**EEF1A1**	10.86 ± 0.88	10.89 ± 2.62	10.88 ± 1.89	12.34 ± 0.75	12.90 ± 1.39	12.62 ± 1.08
**H3F3A**	15.90 ± 0.65	16.34 ± 0.83	16.12 ± 0.76	18.35 ± 0.91	19.15 ± 1.72	18.75 ± 1.34
**YWHAZ**	19.90 ± 2.09	19.92 ± 2.65	19.91 ± 2.32	15.85 ± 0.43	15.30 ± 0.36	15.58 ± 0.47
**PPIA**	-	-	-	15.77 ± 0.33	16.18 ± 0.23	15.98 ± 0.34

**Fig 2:**
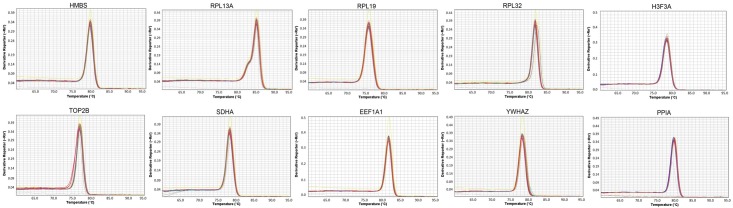
Melting curve analyzes of the 10 reference candidate genes evaluated in this study.

Most of the genes started the amplification between cycles 10 to 20 cycles (Fig 1), indicating high levels of expression. Also, it was possible to identify a higher dispersion of the Cts for the *YWHAZ* gene in E1 compared to E2. According to the melting curve analysis, all genes presented a specific amplification (Fig 2).

**Fig 3:**
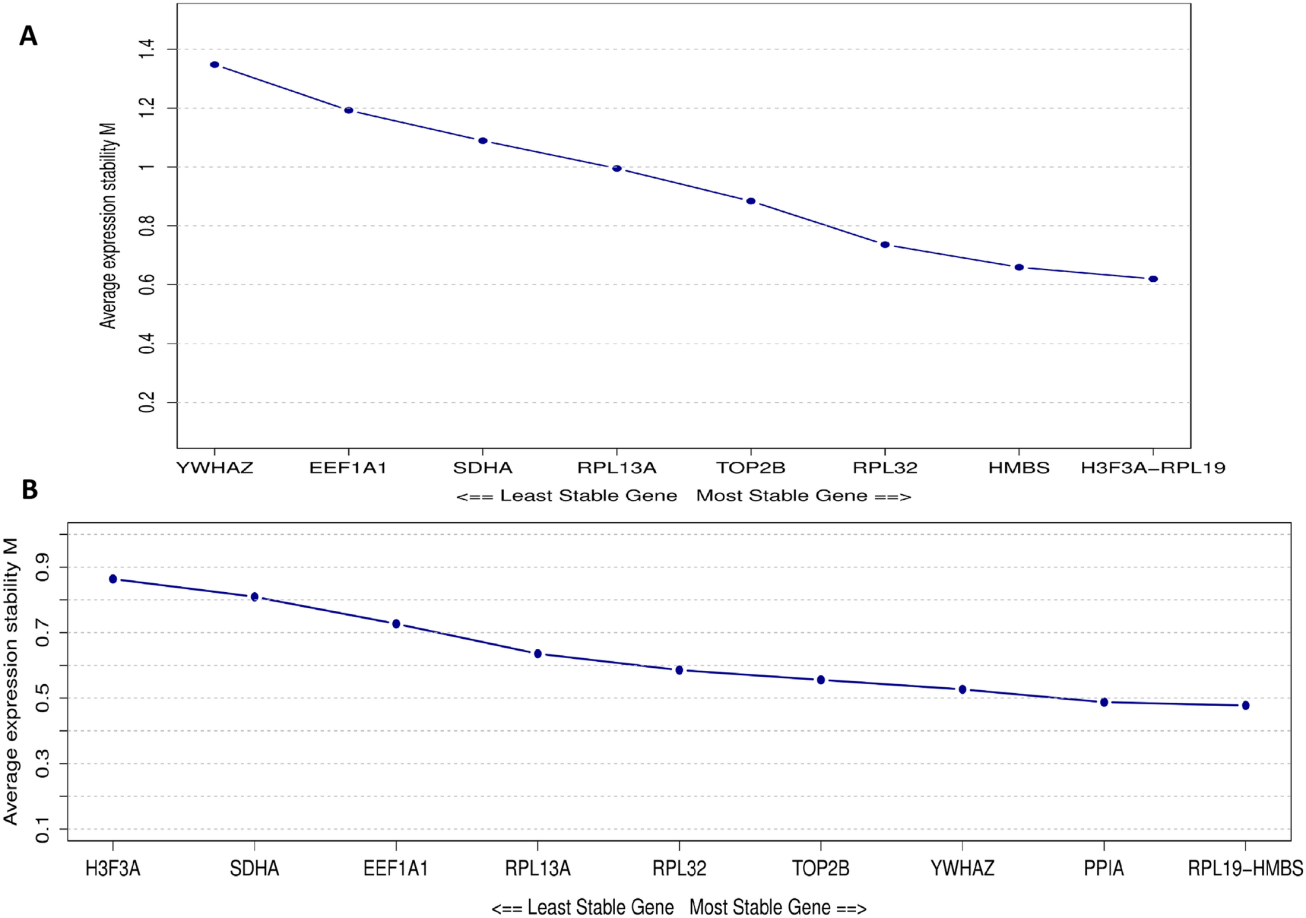
Ranking of reference candidate genes based on the average expression stability using the geNorm software. A: results obtained in the Experiment 1. B: results obtained in the Experiment 2.

Regarding the experiment 1, it was possible to observe a similar expression profile among *RPL19*, *RPL32*, *H3F3A* and *HMBS* genes obtained with the several evaluated tools (Table 3). These were the first four genes ranked with the BestKeeper (S1 Table) and with the geNorm (Fig 3A) tools and also presented the smallest dispersion of Ct values (Fig 1). The geNorm classified the *RPL19 / H3F3A* and *HMBS* genes with the lowest M values: M = 0.620 and M = 0.659, respectively (Fig 3A). The NormFinder program included the *TOP2B* among the most stable genes, while BestKeeper, geNorm and DeltaCt ranked those genes in the last five positions, showing a reduced stability (Table 3). Another important observation was that the *RPL13A*, *SDHA*, *EEF1A1* and *YWHAZ* genes demonstrated the lowest stability values according to the BestKeeper, geNorm and NormFinder tools (Table 3). However, when the ΔCt comparative approached was evaluated, the *YWHAZ* gene was scored as the most stable, differing from the other three tools previously mentioned (Table 3).

**Table 3:** Gene classification values and ranking (in parenthesis) according to the four algorithms analyzed and the general rank generated by the BruteAgreeg for experiments 1 (E1) and 2 (E2). 1^st^ and 2^nd^ are the rank after running the BruteAgreeg twice.

Gene	BestKeeper Power of the gene		DeltaCt Mean StdDev		NormFinder S-value		geNorm M-value		BruteAgreeg			
									E1		E2	
	E1	E2	E1	E2	E1	E2	E1	E2	1^st^	2^nd^	1^st^	2^nd^
HMBS	1.334 (1)	0.000 (1)	1.202 (4)	0.811 (5)	0.542 (4)	0.205 (4)	0.659 (3)	0.478 (2)	3	3	3	4
RPL19	1.386 (2)	1.242 (4)	1.145 (2)	0.796 (4)	0.477 (2)	0.220 (6)	0.620 (2)	0.478 (1)	**1**	**1**	**2**	**2**
H3F3A	1.393 (3)	5.416 (10)	1.214 (5)	1.081 (10)	0.551 (5)	0.270 (9)	0.620 (1)	1.097 (10)	4	**2**	10	10
RPL32	1.632 (4)	1.549 (6)	1.157 (3)	0.871 (6)	0.434 (1)	0.192 (3)	0.736 (4)	0.646 (6)	**2**	4	6	6
TOP2B	2.244 (5)	0.963 (2)	1.259 (6)	0.909 (8)	0.498 (3)	0.321(10)	0.884 (5)	0.599 (5)	5	5	7	7
RPL13A	2.325 (6)	2.485 (7)	1.338 (7)	0.724 (3)	0.633 (6)	0.118 (1)	0.995 (6)	0.761 (7)	6	6	5	5
SDHA	2.351 (7)	4.652 (9)	1.376 (8)	1.023 (9)	0.660 (7)	0.264 (8)	1.089 (7)	1.081 (9)	7	7	9	9
EEF1A1	2.742 (8)	4.002 (8)	1.584 (9)	0.897 (7)	0.900 (8)	0.217 (5)	1.192 (8)	1.001 (8)	9	9	8	8
YHWAZ	3.204 (9)	1.225 (3)	1.141 (1)	0.715 (2)	1.175 (9)	0.238 (7)	1.348 (9)	0.566 (4)	8	8	4	3
PPIA	-	1.422 (5)	-	0.701 (1)	-	0.218 (2)	-	0.492 (3)	-	-	**1**	**1**

When the analysis was performed in the experiment 2, several differences among the most suitable genes were found in comparison to experiment 1. Also, it is interesting to note that each algorithm/tool indicated one different gene as most stable (Table 3). Using geNorm, the *RPL19/HMBS* and *PPIA* genes presented the lowest M value, of 0.478 and 0.492, respectively, which suggest that those genes should be used as reference in E2 (Table 3, Fig 3B). The best genes according to BestKeeper were *HMBS*, *TOP2B* and *YHWAZ* (Table 3), respectively, while the *RPL13A*, *RPL32* and *PPIA* genes were listed by NormFinder. Furthermore, for the ΔCt method, the *PPIA*, *YHWAZ* and *RPL13A* were the top three reliable genes (Table 3). The variation in the stability could occur by differences in the biological expression levels and also due to technical issues. Although all the evaluated primers were specific, a small “shoulder” in the *RPL13A* melting curve (Fig 2) was observed, possibly indicating the amplification of more than one *RPL13A* isoform. This could influence the observed Ct levels, however, since all the samples had the same shoulder and the quantification is relative, probably this shoulder has slightly or no influence on the results.

**Fig 4:**
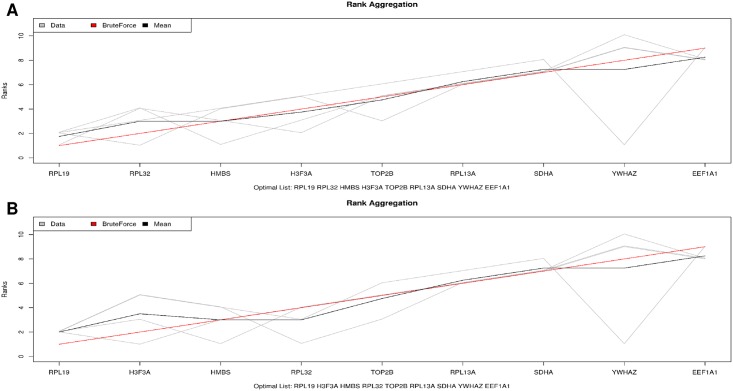
Suitable genes ranked by the BruteAgreeg tool in the two simulations for Experiment 1. A: simulation 1, genes *RPL19*, *RPL32* and *HMBS*; B: simulation 2, genes *RPL19*, *H3F3A* and *HMBS* (Table 3).

**Fig 5:**
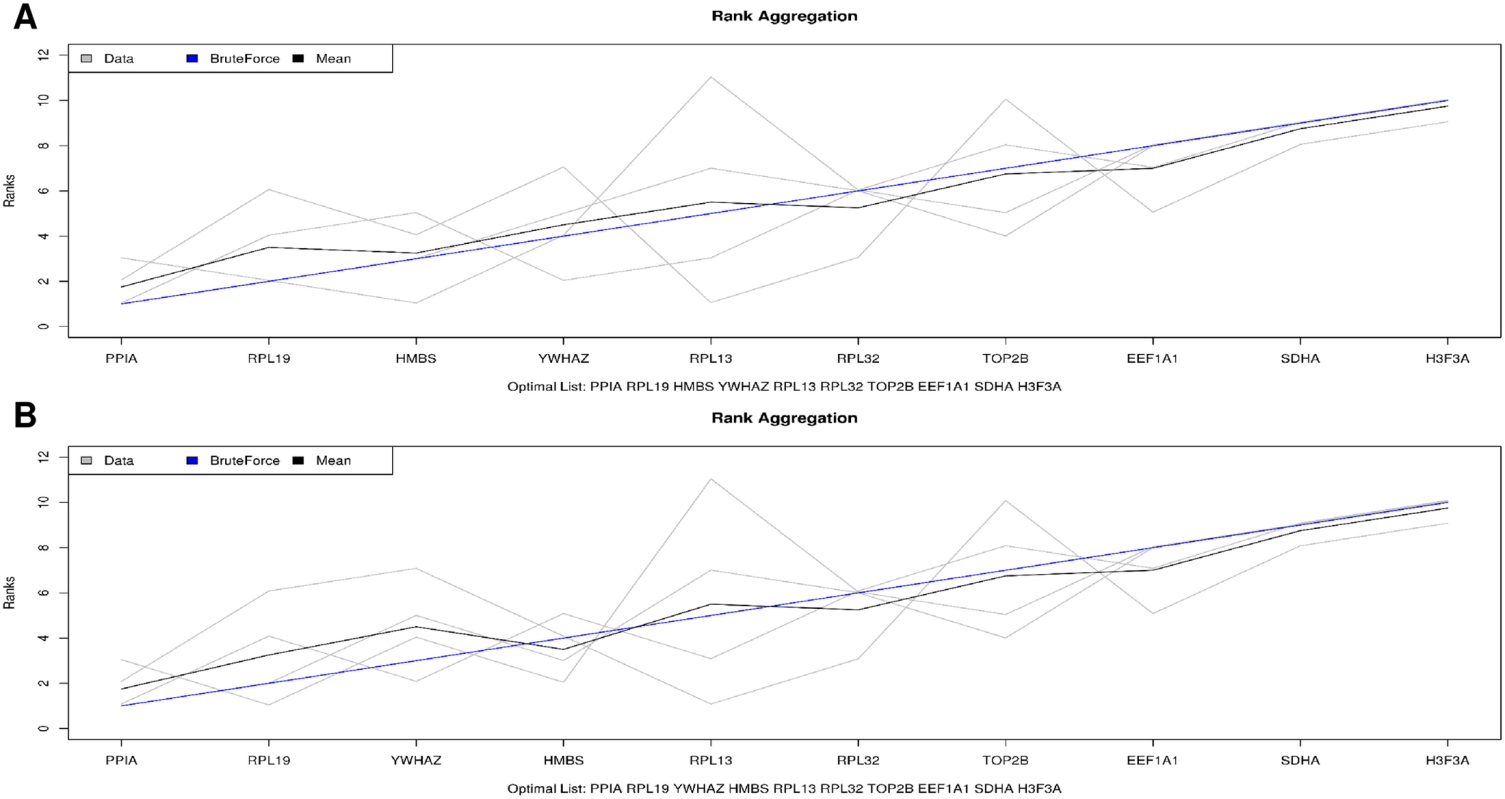
Suitable genes ranked by the BruteAgreeg tool in the two simulations for Experiment 2. A: Simulation 1, genes *PPIA*, *RPL19* and *HMBS*; B) Simulation 2, genes *PPIA*, *RPL19* and *YWHAZ* (Table 3).

A great variation on the rank of the best reference genes were observed, depending on the evaluated tool. Therefore, a general rank considering those four tools was performed using the BruteAggreg function. For the experiment 1, *RPL19* and *RPL32*, and *RPL19* and *H3F3A* were pointed out as the first and second most stable genes, followed by *HMBS*, after performing the BruteAggreg function twice (Fig 4A and 4B, respectively). The results from the BruteAggreg function are similar to those obtained with the geNorm evaluation, including for the genes *EEF1A1* and *YWHAZ*, which were the worst genes evaluated (Fig 4). For the experiment 2, the *PPIA* and RPL19 were scored as the best genes in both BruteAggreg analyses, while the H3F3A and SDHA were the most variable genes (Fig 5A and 5B, respectively).

The *RPL19* was ranked as one of the less variable genes, showing a similar classification (Table 3) for both experiments, which were run independently. Also, the *HMBS* gene was classified as the 3^rd^ most stable in the general rank for E1 and the 3^rd^ and 4^th^ for E2 (Figs 4 and 5). However, despite of these similarities, there were two important differences in the general score: one related to the *H3F3A* gene, that was the 2^nd^/4^th^ most stable gene in experiment 1, but was the worst gene evaluated in the experiment 2, and the *PPIA* gene, which was the best gene to be considered as reference in the experiment 2, while several samples did not amplify in the experiment 1.

## Discussion

The studies using gene expression methodologies have been increasing and the use of qPCR for mRNA quantification might be highlighted [32]. Although the qPCR analysis is widely disseminated, some concerns are always important to improve the quality of the laboratory analyses. One of them is related to the RNA amount and integrity, which helps in achieving high accuracy, sensitivity and reproducibility of the further analysis. In this study, the total RNA with good quality according to the usually recommended [32,33] and an amount necessary for all the expression analysis in just one batch was obtained.

The evaluation of a large set of reference candidate genes is essential to obtain reliable data in qPCR studies [28,34]. For this purpose, 10 putative reference genes were evaluated according to their expression stability and consistency with four different specific tools: geNorm, NormFinder, BestKeeper and ΔCt method (Table 3), which are widely used in similar studies. In the last years, several studies have been published discovering many candidate genes that might be used as internal control [28,34]. However, the search for the best reference gene is not trivial, since there are many approaches available and no standard methodology is established. In addition, some of the algorithms include a threshold stability value, while others do not and these values are helpful to verify if the top genes are indeed stable. Therefore, the use of those tools jointly provides more information on the variability of gene expression, which improved our decision on choosing the most reliable reference genes among the studied candidates. Moreover, each experiment/condition requires a specific search for genes with non-variable expression patterns to be used as control [34].

In this study, two independent experiments were carried out in pigs from two different lines and ages. In general, it was possible to observe a discordance of the best normalizer genes chosen among the four methodologies in both experiments for the inguinal ring tissue (Table 3). These results reinforce the need for checking a certain number of reference candidate genes before initiating a gene expression analysis, in order to have an appropriate normalization of the transcript level [35].

In pigs, there are some studies validating internal control genes in multiple tissues, such as backfat, muscle, heart, adipose, skin, liver, pancreas, lung, within others [10,20,24] and also, in various developmental stages [22]. Although several tissues have already been evaluated, no information about expression profile of the inguinal ring has been reported to date, especially considering the presence or absence of scrotal hernia phenotype.

In our study, 10 reference candidate genes were tested. However, some issues to select the best genes were encountered, since the most stable reference genes varied when each tool was evaluated separately. For example, while the *HMBS* was the best ranked in the BestKeeper in both experiments, it was the 2^nd^ and 3^rd^ in the geNorm (Fig 3), the 4^th^ and 5^th^ for Delta Ct, and 4^th^ in the NormFinder, for E1 and E2, respectively. Other studies, such as the one reported by Perez, Tupac-Yupanqui & Dunner (2008) [36], also found a divergent pattern among the tools evaluated for internal control genes in bovine muscle tissues. Obviously, this variation could happen, since the algorithms and data transformation of those tools are different and were developed to address different types of experiments. While the geNorm uses an arithmetic mean of all pairwise variation to obtain the M value and do not account for co-expression of the reference candidate genes, ranking the 2 most stable reference genes [26], the NormFinder uses a model-based approach generating the S value, having low sensitivity to the co-regulation of the genes [27]. In addition, Bestkeeper allows the visualization of raw and transformed data and generates a combined index of the best genes, being sensitive to those with large differences in their Ct levels [28]. While a similar amount of mRNA are required to the use of the previous tools, the comparative DeltaCt bypasses this requisite, being advantageous for experiments with limited amount of input RNA [29]. Therefore, when such a great variation is observed on the genes ranked by different tools, there is no recommendation of the best method to select the genes, as well as there is no standard score or threshold indicating good or bad stability. Mosley et al. (2017) [37], after analyzing 5 tools (BestKeeper, geNorm, NormFinder, DeltaCt and RefFinder), concluded that the geNorm seems to be the best tool for choosing the most reliable genes.

Some studies have generated a rank when several approaches are used as an alternative to choose the best normalizer genes [38–40]. Thus, the validation with another tool is essential to improve the quality of the genes to be chosen [41]. Therefore, a general ranking obtained with the BruteAggreg function pointed out that for the E1, *RPL19*, *H3F3A/RPL32* and *HMBS* (Fig 4) were the most stable genes, while for the E2 the most stable genes were *PPIA*, *RPL19* and *HMBS/YWHAZ* (Fig 5, Table 3). Regarding gene stability, the top genes in the general rank were also considered stable genes in most of the tools separately, since they had values within the parameters suggested by each tool, i.e. S < 0.5, M < 1.5 and [±Ct] SD < 1.5 (Table 2, S1 Table). Also, a similar pattern was observed when the least stable genes were ranked, i.e., the *YHWAZ* in E1, which was considered unstable in the NormFinder (S = 1.175) and Bestkeeper ([± Ct] SD = 1.87, S1 Table) tools and the most variable in the geNorm (M = 1.348). Therefore, the use of several tools to choose the normalizer gene(s) allowed us to verify the variation on the expression of those reference candidate genes in a widely way. Even though BruteAggreg provides a general rank of the genes, this does not mean that all genes are stable or vice-versa. In this context, there is the need to evaluate the output from different tools according to their stability values to consider if the genes are indeed stable or not. The *H3F3A* and *HMBS* have been previously described as reference genes in swine tissues, where the *H3F3A* was the most stable and the *HMBS* was regulated in some of the evaluated tissues [23,24]. Few studies have been performed using the *RPL19* as reference gene in pigs [42], but it has been considered as a good internal reference gene in other livestock species [43–46]

Regarding the best endogenous genes for the inguinal ring tissue, the *RPL19* showed the highest uniformity in its expression within the tools and experiments (Table 3, Table 2). Ribosomal proteins have been suggested as good reference genes in many studies [47], because of their function on ribosome production [48]. Schulze et al. (2017) [45] and Lenart, Kogut & Salinska (2017) [46] also found stable expression of this gene on sheep bone cells and in chick brain, respectively. In pigs, *RPL19* was recommended as endogenous gene in studies using peripheral blood mononuclear and dendritic cells [42]. The *RPL19* amplified in early Cts (before 20) and had small coefficient of variation in each experiment (Fig 1, Table 2), which can indicate that this gene would be a good normalizer. The *RPL32*, *H3F3A* and *RPS18* (ribosomal protein 18S), involved in the development of cellular machinery, have also been chosen as endogenous gene for multiple tissues and swine breeds [14,23]. In addition, Zhang et al. (2012) [23], testing six endogenous genes in the *longissimus dorsi* of pigs, found differences on the best genes according to the breeds studied, where *RPL32* / *RPS18* were the most stable in the Landrace and *H3F3A / RPS18* in the Toncheng breed. In our study, both *RPL32* and *H3F3A* genes were the 2^nd^ most stable genes in the E1 (Table 3, Fig 4), endorsing the results obtained by Zhang et al. (2012) [23]. On the other hand, for the E2, the *RPL32* and *H3F3A* were not considered as stable genes.

The *PPIA* gene, that is involved in protein folding [49], has also been recommended for being used as endogenous control in several tissues, species and ages [9,14,21]. In our study, the *PPIA* was ranked as the most invariable gene in the E2, where Landrace pigs with 60 days of age were evaluated, being one of the most indicated as endogenous gene from the geNorm, NormFinder and Comparative Ct tools (Table 3, Fig 3). A similar pattern was observed when several tissues of Berskshire, Duroc, Landrace and Yorkshire pigs were evaluated [14], suggesting that *PPIA* is a reliable gene for expression studies in adult pigs. However, in our study, a variation in the *PPIA* expression between the two experiments was observed (Table 3). Although the *PPIA* was the best normalizer gene in the E2, for E1, in which the samples were obtained from 30 days-old MS115 pigs, the expression of this gene was impossible to be analyzed, since many samples did not amplify. This might be due to the different ages and breeds used in each experiment. Uddin et al. (2011) [9] observed that distinct genes should be used as reference gene depending on the pig’s age. In addition, stability differences can also occur among the tissues analyzed [12]. Here, samples of the inguinal ring were collected and, albeit a unique technician had collected all samples in a specific anatomical region, it could be possible that the tissues were slightly unequable among samples and experiments, since this tissue is highly complex to collect. Therefore, studies evaluating the distinction among breeds, phenotypes and age of the animals are essential [9] to the better characterize the expression profile of the tissues.

The *HMBS* gene had a good general ranking and in most of the other tools for both experiments (Table 3). This gene has been used as endogenous in many species, pig lines, tissues and ages [24,38,50,51]. However, the regulation of this gene depends on the muscle tissue, sex, age and experimental conditions [38,50].

The *H3F3A* and *YWHAZ* were the most variable genes between both experiments. For instance, *H3F3A* was considered reliable in the experiment 1, with 30 days-old MS115, while it was the least reliable in the experiment 2, with 60 days-old Landrace pigs. The same pattern was observed with the *YWHAZ*, which in this case was stable with 60 days-old Landrace samples and variable with the 30 days-old MS115, reinforcing the statement that there are no general reference genes that might be used in all situations. The *SDHA*, *TOP2B*, *EEF1A1* and *YWHAZ* genes were highly variable regarding the general score in both experiments, possibly because of the late Ct and its variation between and within groups. Furthermore, the variability presented by these genes could be possibly due to the non-homogeneity of the tissue used in this study.

Although more than two genes should be used as reference in gene expression studies [33], the average number of genes used is only 1.2, which means, below the recommendation [33,34]. Moreover, it is usual studies with relatively common genes such as GAPDH, β-actin and 18S RNA, without testing for stability. Given the complexity of the experimental designs and tissues to be evaluated, a broad panel of genes and tools should be used to search for the best reference genes [34]. It is also important to note that when candidate reference genes are being evaluated, the most or least stable genes chosen are based only in that experiment, and not necessarily will happen in other conditions. Furthermore, the most stable genes found in one experiment does not mean that only those genes are stable, reinforcing the need of always testing several candidate reference genes. The use of more than three genes is indicated to reduce the selection of false endogenous genes that may impact on the reliability of the results [52]. One example could be observed in our study, where the same tissue was collected from animals of two different lines and ages and, despite of being from the same species, two sets of genes should be used as reference: the *RPL19*, *RPL32 and H3F3A* for 30-days MS115 (E1) and *PPIA* and *RPL19* for the 60 days-old Landrace pigs (E2).

In this study, even though there was a confounding between age and breed effects, the experimental conditions influenced the stability of the evaluated genes. Therefore, further studies are recommended to clarify the isolated contribution of age and breed to variations on the genes’ expression profile in the inguinal ring tissue of pigs. The effect of breed is expected to influence scrotal hernia congenital anomaly. Vogt & Ellersieck (1990) [18] found significant differences in frequency of this defect among Duroc, Landrace and Yorkshire male lines. Sevillano et al. (2015) [19] observed a slightly higher incidence of scrotal hernia in Large White (0.42%) compared to Landrace breeds (0.34%). In addition, these authors mapped distinct genomic regions associated to scrotal hernia between Landrace e Large White pigs. Probably, intrinsic conformation and anatomical differences of each breed could affect the inguinal ring tissue composition causing variation in the expression profile of the endogenous candidate genes. Regarding the age effect, since hernias are related to development, usually resulting from failed obliteration of the *processus vaginalis* after descent of the testis, it is expected that the age would be important to this malformation. As evidence, most scrotal hernias are diagnosed at the time of castration, an early phase in the pig’s life [53]. Therefore, the age effect should be evaluated independently in different ages, especially in early stages of life.

## Conclusions

The breed/age effects influenced the expression stability of candidate reference genes evaluated in the inguinal ring of pigs. A consensual set of reference genes was not obtained for the two experimental conditions, evidencing the importance of evaluating the stability of several endogenous genes previous their use. The *RPL19* was one of the most reliable endogenous genes for both experiments. Therefore, two set of genes are recommended for the inguinal ring tissue: *RPL19*, *RPL32* and *H3F3A* for 30-days MS115 and *PPIA* and *RPL19* for the 60 days-old Landrace pigs. This is the first study using the inguinal ring tissue and the results can be useful as an indicative for other studies working with gene expression in this tissue.

## Supporting information

S1 Table Results from the Bestkeeper tool. (XLSX)
